# Effects of perioperative dexmedetomidine on renal vascular function and renovascular histopathology in ovine cardiopulmonary bypass

**DOI:** 10.1186/s40635-025-00836-7

**Published:** 2025-12-16

**Authors:** Ashenafi H. Betrie, Alemayehu H. Jufar, Roger G. Evans, Andrew D. Cochrane, Bruno Marino, Ian Birchall, Sally G. Hood, Peter R. McCall, Scott Ayton, Lachlan F. Miles, Clive N. May, Yugeesh R. Lankadeva

**Affiliations:** 1 Florey Institute of Neuroscience and Mental Health, The University of Melbourne, Victoria, Australia; 2 Florey Department of Neuroscience and Mental Health, The University of Melbourne, Victoria, Australia; 3 Biomedicine Discovery Institute and Department of Physiology, Monash University, Melbourne, VIC Australia; 4 Department of Cardiothoracic Surgery, Monash Health and Department of Surgery, Monash University, Melbourne, VIC Australia; 5 Cellsaving and Perfusion Resources, Melbourne, VIC Australia; 6 Department of Surgery, The University of Melbourne, Victoria, Australia; 7 Department of Anaesthesia, Austin Hospital, Melbourne, Australia; 8 Department of Critical Care, Melbourne Medical School, The University of Melbourne, Victoria, Australia; 9 Translational Cardiovascular and Renal Research Group, The Florey Institute of Neuroscience and Mental Health, 30 Royal Parade, Parkville, VIC 3052 Australia

**Keywords:** Cardiopulmonary bypass, CPB, Dexmedetomidine, Acute kidney injury, Renal interlobar arteries, Vasoconstriction, Sheep

## Abstract

**Background:**

Cardiopulmonary bypass (CPB) is integral to the conduct of cardiac surgery but is associated with postoperative acute kidney injury (AKI). Dexmedetomidine, an α₂-adrenoceptor agonist with anti-inflammatory and sympatholytic properties, has putative renoprotective effects. In a recent meta-analysis, dexmedetomidine during CPB reduced AKI; conversely, a large, randomised trial reported an increase in postoperative AKI. Further, we found increased renal tubular injury in sheep receiving dexmedetomidine during CPB. Here, we aimed to determine whether dexmedetomidine during CPB induces changes in renal vascular reactivity or endothelial integrity that could explain focal renal tubular injury.

**Methods:**

Fourteen instrumented Merino ewes underwent 2 h of non-pulsatile CPB (flow 70 mL/kg/min; MAP 65–75 mmHg; cooled by 3 °C) under standardised propofol–fentanyl–sevoflurane anaesthesia. Animals were randomly allocated to dexmedetomidine (0.4–0.8 µg/kg/h, *n* = 7) or fluid-matched saline (*n* = 7) from induction of anesthesia to end-CPB. Arterial pressure, renal blood flow, cortical and medullary perfusion and PO₂ were measured in vivo (*n* = 7/group). Post-CPB, renal interlobar arteries were isolated for wire myography. Due to standardisation failures, in vitro analyses of dose–response curves for phenylephrine were performed in *n* = 6 per group, while endothelial-dependent and independent relaxation responses were performed in *n* = 7 per group. Endothelial histology of CPB arteries was compared with arteries from a separate cohort of healthy Merino ewes (*n* = 7).

**Results:**

In vitro functional investigations demonstrated that interlobar arteries from dexmedetomidine-treated sheep exhibited a 2.3-fold increase in phenylephrine sensitivity (*p*EC₅₀ 5.82 ± 0.27 vs. 5.45 ± 0.23; *p* = 0.034), with unchanged maximal contraction. Endothelium-dependent and independent relaxations were similar between groups, though inhibitor studies indicated a shift towards cyclooxygenase-mediated dilation under dexmedetomidine. Histology revealed intact endothelial architecture and no damage to endothelial integrity in all groups.

**Conclusions:**

Perioperative dexmedetomidine during CPB enhanced α₁-adrenergic vasoconstrictor sensitivity in renal interlobar arteries without disrupting endothelial integrity or compromising renal blood flow or intrarenal perfusion. The enhanced vasoreactivity may contribute to focal renal ischaemia and tubular injury during CPB, which cannot be detected by in vivo measurements of global and regional kidney perfusion and oxygenation. Further investigation is warranted to elucidate the pathways through which dexmedetomidine contributes to renal tubular injury during CPB.

**Supplementary Information:**

The online version contains supplementary material available at 10.1186/s40635-025-00836-7.

## Introduction

Cardiac surgery requiring cardiopulmonary bypass (CPB) is one of the most important medical developments of the twentieth century, and is essential for the safe conduct of open heart surgery. [1] However, despite its many benefits, a significant percentage of patients develop acute kidney injury (AKI) after CPB [2–4].

Dexmedetomidine is a selective alpha 2 (α_2_)-adrenoceptor agonist that is used to manage patients in intensive care units; it has sedative, analgesic, anxiolytic, anti-inflammatory and sympatholytic properties [5–7]. Various clinical trials have assessed whether dexmedetomidine decreases the incidence of cardiac surgery-associated AKI [8]. A meta-analysis of 16 studies that included 2148 patients reported that dexmedetomidine reduced the incidence of AKI and length of stay in the intensive care unit after cardiac surgery [9]. However, a recent randomised controlled trial of perioperative dexmedetomidine in 652 adults undergoing CPB reported a significantly higher incidence of postoperative AKI [10], providing equipoise for a more detailed investigation into its effects on the kidneys. We recently demonstrated that perioperative dexmedetomidine was associated with a higher prevalence of renal tubular injury in sheep undergoing CPB. However, no plausible explanation for this signal of renal harm was found: measurements of total renal blood flow and renal cortical and medullary perfusion and oxygenation were unaffected by dexmedetomidine treatment during CPB in sheep in our experiments [11]. This current study was performed to assess the direct renal vascular effects of dexmedetomidine.

Intravenous dexmedetomidine has direct vasoconstrictor effects in various peripheral vascular beds across multiple species: in ovine sepsis [12, 13]; in human hand veins [14]; in internal mammary arteries [15]; and in dog coronary circulation [16, 17]. There is a paucity of data on the combined effects of CPB and perioperative dexmedetomidine on vascular reactivity and histopathology in isolated small resistance renal arteries. We therefore performed a placebo-controlled, randomised preclinical study in sheep to test whether perioperative dexmedetomidine impairs renal vascular reactivity to vasoconstrictors and vasodilators and endothelial integrity. We hypothesised that the renal tubular injury associated with dexmedetomidine during CPB, which we reported previously [11], is due to impaired renal vascular reactivity and endothelial integrity. Our primary outcome was the change in renal vascular contraction to dexmedetomidine that could lead to hypoperfusion in the renal microcirculation.

## Methods

### Animals

All animal experimental procedures were performed in accordance with the Australian code for the care and use of animals for scientific purposes and were approved by the Florey Institute of Neuroscience and Mental Health Animal Ethics Committee (18-119-FINMH). All studies fulfilled the Animal Research: Reporting of In Vivo Experiments (ARRIVE) and ARRIVE 2.0 criteria [18, 19].

Fourteen female non-pregnant Merino ewes (40–46 kg) were housed in individual metabolic cages with free access to water and 800 g of oaten chaff daily and were allowed a week of acclimatisation to the laboratory environment. This study was done contemporaneously with our previously reported ovine study assessing the effects of dexmedetomidine on the renal macro- and micro-circulation in vivo [11]. Twelve of the 16 sheep from our previously reported study and an additional 2 sheep that were not included in the previous study were used to isolate small resistance interlobar arteries in this in vitro study. At the end of 2 h of CPB following aortic cross-clamp, sheep were euthanised and the renal interlobar arteries were isolated for functional and histopathological assessment. A group of healthy sheep (*n* = 7) were euthanised as controls for histopathological assessment of renal interlobar arteries.

### Study design

The surgical procedures, CPB conditions and detailed methodology have been detailed previously [11]. Briefly, sheep underwent an initial surgical procedure to cannulate the jugular vein, renal vein and carotid artery, to implant a transit-time flow probe (4 mm; Transonic Systems, Ithaca, NY) around the left renal artery and custom-built fibre optic probes (Oxford Optronix, Abingdon, United Kingdom) in the left kidney, and to insert a Foley catheter into the bladder [11, 20]. Following surgery, analgesia with intramuscular flunixin meglumine (50 mg; Norbrook, Tullamarine, Victoria, Australia), and procaine penicillin (900 mg; Troy Laboratories, Glendinning, NSW, Australia), was given for 3 days.

**Fig. 1:**
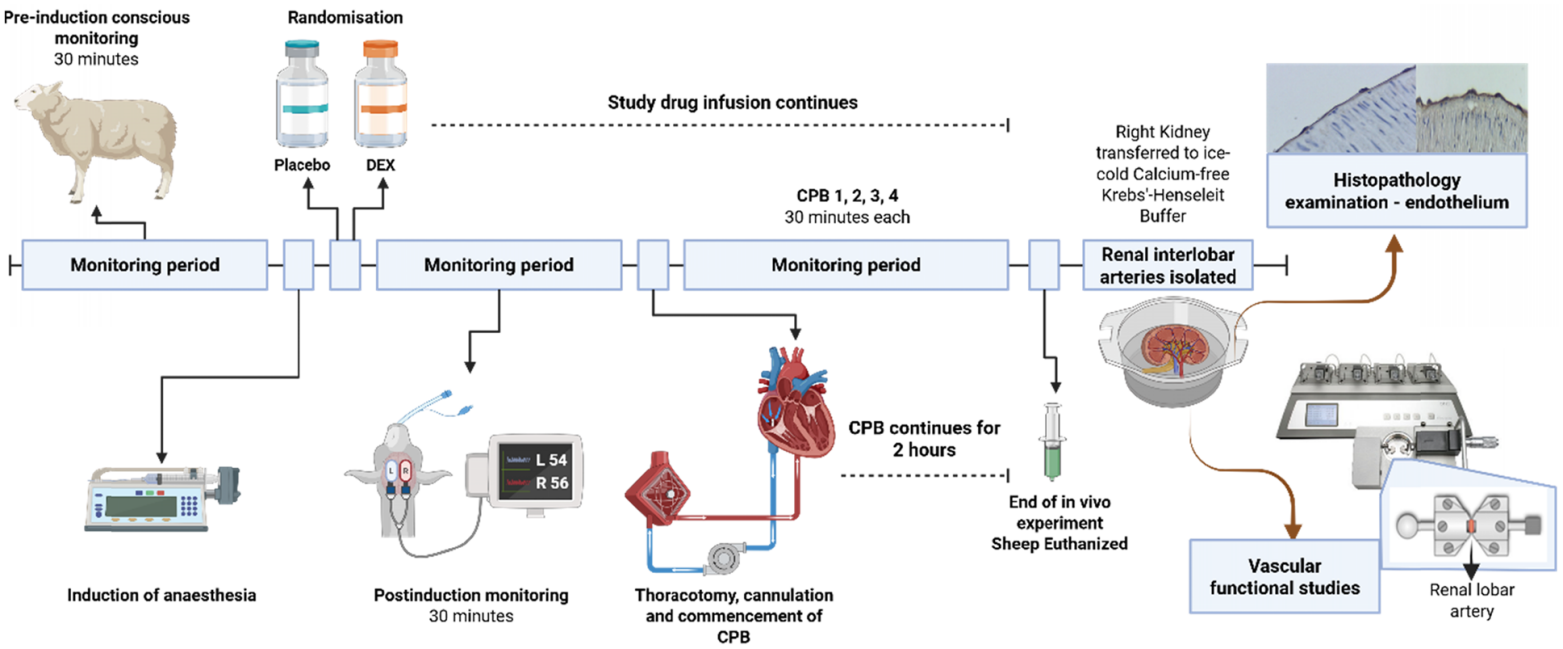
Study schematic of the ovine dexmedetomidine in vivo cardiopulmonary bypass study followed by the in vitro functional/histopathology study

After 5 days of recovery, sheep were randomised to receive dexmedetomidine (*n* = 7) or volume-matched saline (*n* = 7). Researchers conducting the experiments were not blinded to group allocation. Animals underwent six 30-min recording periods: conscious baseline, after induction of anaesthesia and four 30-min intervals after achieving full flow on CPB (Fig. 1). Results from the conscious state, under anaesthesia and the final 30-min of a 2-h CPB recording are reported. Arterial pressure, renal blood flow, renal cortical and medullary tissue perfusion, tissue oxygen tension (PO_2_) and temperature were recorded.

### Cardiopulmonary bypass and intervention

Sheep received intravenous propofol (4 mg/kg, AFT Pharmaceuticals, Burwood, NSW, Australia) and fentanyl (5 µg/kg, Hameln Pharmaceuticals, Hameln, Germany) to induce anaesthesia followed by inhaled sevoflurane (4%, AbbVie, Mascot, NSW, Australia) and intravenous propofol (4 mg/kg/h) and fentanyl (3 µg/kg/h) as maintenance. Intravenous compound sodium lactate (2 ml/kg/h, Baxter, Toongabbie, NSW, Australia) was used as maintenance fluid. Sheep randomly allocated to the dexmedetomidine group received a continuous infusion of 0.4 µg/kg/h dexmedetomidine (PRECEDEX™, Pfizer, Hospira, Melbourne, VIC, Australia) after anaesthesia was induced (after stable haemodynamic status was established), followed by 0.8 µg/kg/h after CPB commenced, as described previously [11], to mirror a clinically acceptable dosing regimen to and avoid bradycardia. CPB was established, targeting a non-pulsatile pump flow of 70 ml/kg/min, MAP of 65–75 mmHg and 36 °C core body temperature (3 °C below normal body temperature for sheep) [20–22].

### Functional in vitro protocols in isolated renal arteries

At the end of 2 h of CPB, sheep were euthanised with intravenous sodium pentobarbitone (200 mg/kg, Virbac, Wetherill Park, Australia). The right kidney was removed immediately postmortem, cut in half longitudinally and pinned out on a Silastic-bottomed petri dish filled with ice-cold physiologic salt solution (PSS) with the following composition (mM): NaCl 119; KCl 4.69; MgSO_4_.7H_2_O 1.17; KH_2_PO_4_ 1.18; glucose 5.5; NaHCO_3_ 25; saturated with carbogen (FO_2_ 95%; FCO_2_ 5%) at pH 7.4.

Renal interlobar arteries (250–450 µm internal diameter, i.d.) were dissected from the surrounding tissue and segments of arteries (~ 2 mm length) were mounted in separate myograph chambers (Model 610 M and 620 M; Danish Myo Technology, Denmark) containing PSS with 2.5 mM CaCl_2_ at 37 °C for isometric force measurement [23–25]. Contractile responses were recorded with LabChart 7 and a PowerLab 4/30 A/D converter (AD Instruments Pty Ltd, Australia). To normalise the basal conditions, the vessels were stretched passively to a diameter setting of 90% of that determined to be an equivalent transmural pressure of 100 mmHg. After allowing the tissues to equilibrate for 30 min, the arteries were exposed to a potassium depolarising solution (100 mM K^+^ replacing Na^+^ in PSS; termed KPSS) and phenylephrine (10 µM) for 2 min. A second exposure to the KPSS solution was used to provide a reference contraction. The presence of functional endothelium was tested by initially contracting the arteries sub-maximally (80% of KPSS contraction) with phenylephrine and/or U46619, a thromboxane mimetic followed by a single concentration of the endothelium-dependent relaxant, bradykinin (1 µM). Only arteries that gave more than 50% relaxation to bradykinin were considered to have intact endothelium.

Contractile responses were assessed by performing cumulative response curves to phenylephrine (0.1–100 µM). For relaxation experiments, an initial stable precontraction was obtained using a combination of U46619 and/or phenylephrine before completing response curves to sodium nitroprusside (0.001–1 µM; endothelium-independent relaxation) or bradykinin (0.01–1000 nM; endothelium-dependent relaxation), To assess changes in the contribution of nitric oxide and vasodilatory prostanoids to the relaxation by bradykinin, a separate group of arteries was preincubated with the nitric oxide synthase inhibitor, N^ω^-nitro-L-arginine-methyl ester (200 µM, L-NAME), or a combination of L-NAME and the cyclooxygenase inhibitor, indomethacin (3 µM) for 30 min before performing relaxation response curves to bradykinin (0.01–1000 nM).

### Histopathological assessment of isolated renal arteries

A subset of ovine fresh renal interlobar arteries was isolated and fixed in 10% neutral buffered formalin. Arteries from healthy sheep (*n* = 7) were collected as controls. The arterial segments were processed using standard histological protocols and embedded in paraffin [26]. Longitudinal and cross-sectional serial sections (5 µm) through each artery were prepared and stained with haematoxylin and eosin (H&E). Endothelial morphology was demonstrated with a biotinylated, ovine endothelial cell specific lectin, *Datura stramonium* L (Vector B-1185). The lectin was diluted 1/50 in 0.1 M Tris buffer and applied for 1 h followed by a 20-min incubation with peroxidase conjugated streptavidin (Agilent K0675). Positive labelling was detected with 3,3, diaminobenzidine (DAB) (Agilent K3467).

Changes to endothelial morphology/lining of each arterial segment were assessed based on the presence of normal, continuous flat endothelium and vacuolated endothelial cells. The histopathological assessments were performed by an independent assessor who was blinded to treatment groups and were graded semi-quantitatively as follows: 0 (no change/normal flat and continuous), 1 (mild change, 5–25% of assessed section affected) and 2 (moderate, 26–50% of assessed section affected).

### Outcome

Our primary outcome was increased renal vascular contraction to phenylephrine that could lead to focal tissue ischaemia and tubular injury. Vascular contractility was assessed using isolated renal interlobar arteries mounted in wire myography. We hypothesised that the renal tubular injury associated with dexmedetomidine during CPB that we observed previously [11] was due to impaired renal vascular reactivity and endothelial integrity. Intravenous dexmedetomidine is reported to cause peripheral vascular contraction in human and animal vessels [12, 14–17, 27]. The secondary outcome was loss of endothelial integrity and function. Endothelial integrity and morphology were assessed under a microscope after H&E or lectin staining.

### Sample size and randomisation

A total of 21 sheep were included. Fourteen sheep underwent CPB and were randomised to dexmedetomidine (*n* = 7) or placebo (*n* = 7). An additional cohort of naïve healthy controls (*n* = 7), euthanised without experimental intervention, was used for histopathological comparison of renal interlobular arteries. All in vivo physiological measurements were obtained from all animals in each CPB group (*n* = 7 per group). For the in vitro vascular reactivity studies, renal arteries were isolated from all animals; however, only 6 arteries per group were included in the final analyses because one artery from each group failed standardisation tests for contractility with phenylephrine using wire myography, likely due to mechanical injury during organ collection or dissection. Endothelial-dependent and independent relaxation to bradykinin and sodium nitroprusside and histology of CPB arteries was compared with arteries from a separate cohort of healthy Merino ewes (*n* = 7). Based on prior data of the mean potency of the α1 agonist, phenylephrine, in healthy ovine isolated renal arteries, a sample size of 5 isolated arteries per group gives a 90% power to detect an 80% change in the potency (relative predicted difference) as a continuous variable. Sheep were allocated to the vehicle or dexmedetomidine group using block randomisation in groups of 4 as previously described [11]. All *in vivo* physiological measurements were obtained from all animals in each CPB group (*n* = 7 per group).

### Statistical analyses

All *in vitro* isolated renal artery data are expressed as mean ± standard deviation (SD), with n being the number of sheep arteries isolated from separate sheep. From each sheep, 8–10 small renal arteries were isolated. Each artery was assigned to a single experimental protocol, resulting in 5–6 distinct protocols performed per sheep. For any given protocol, only one artery per sheep contributed data, and analyses were performed at the sheep level. Thus, *n* represents the number of individual sheep included in each experiment. Each sigmoidal concentration–response curve was fitted using Prism 10 (GraphPad Software, USA). In some cases, the last data point (at the highest concentration) was imputed (replication of the data from the next highest concentration) for better sigmoidal fitting. The *p*EC50 values (the negative log_10_[M] of drug concentration that decreases the response by 50%) and *E*max (maximum response) were determined for each tissue and averaged. Student’s unpaired two-tailed *t*-test was used to analyse differences between the two groups of variables. Two-way ANOVA (or mixed-effects model for missing values) with Sidak’s post-test was used to assess differences within and between groups when 2 or more groups with multiple independent variables are present.

All *in vivo *data are presented as mean ± standard deviation (SD). Data were analysed using a two-way or mixed-effects model (for missing values) repeated measures analysis of variance (ANOVA) with factors ‘group’ (*p*_Group_), ‘time’ (*p*_Time_) and their interaction (*p*_Interaction_). If *p*_Time_, *p*_Group_ or *p*_Interaction_ were ≤ 0.05, Tukey’s post-test was performed to adjust *p* values for making within-group or between-group multiple comparisons at each time point.

For histopathological analysis of the endothelial lining of isolated renal interlobar arteries, comparisons were made using Fisher’s exact test for categorical data or the Mann–Whitney *U* test for ordinal data.

Variances were assessed by the Brown–Forsythe test before performing ANOVA. The adjusted *p* values after individual comparisons (post-test) are reported. Values of *p* ≤ 0.05 (two-tailed) were considered statistically significant.

## Results

The target conditions of MAP (65–75 mmHg) and body temperature (decrease by 3 °C) were achieved during CPB in both groups (Table 1). Total peripheral conductance was similar in the vehicle and dexmedetomidine groups, although we observed increased vascular conductance in the dexmedetomidine group only 1 h after commencing CPB compared with the vehicle control (*p* = 0.034, Table 1). There was a time-dependent increase in systemic oxygen delivery during CPB only in the vehicle control group (*p*_Time_ = 0.003, Table 1). There were no significant differences between the groups at any of the other time points. Systemic oxygen consumption remained comparable within and between groups at all time points (Table 1).

**Table 1:** Effects of dexmedetomidine on arterial pressure, temperature, total peripheral conductance and systemic oxygen delivery and consumption during cardiopulmonary bypass in sheep

Variable	Conscious		Anesthetised		Cardiopulmonary bypass (CPB)								
	Vehicle	Dex	Vehicle	Dex	Vehicle CPB 1	Dex CPB 1	Vehicle CPB 2	Dex CPB 2	Vehicle CPB 3	Dex CPB 3	Vehicle CPB 4	Dex CPB 4	*p*_Interaction_
Mean arterial pressure (mmHg)	89 ± 2	87 ± 7	73 ± 9	72 ± 12	70 ± 3	67 ± 5	71 ± 3	66 ± 3	70 ± 4	68 ± 3	70 ± 4	68 ± 4	0.974
Body temperature (^o^C)	39.5 ± 0.4	39.4 ± 0.2	38.8 ± 0.6	38.9 ± 0.4	36.4 ± 0.3^‡‡‡^	35.9 ± 0.6^‡‡‡^	36.3 ± 0.3^‡‡‡^	36.4 ± 1.1^‡^	36.2 ± 0.4^‡‡‡^	36.1 ± 0.4^‡‡‡^	36.3 ± 0.3^‡‡‡^	36.3 ± 0.1^‡‡‡^	0.471
Total peripheral Conductance (mL/kg/mmHg)					1.00 ± 0.04	1.05 ± 0.08	0.98 ± 0.04	1.06 ± 0.04	1.00 ± 0.06	1.03 ± 0.05	1.00 ± 0.06	1.04 ± 0.07	0.555
Systemic oxygen delivery (mL O_2_/kg/min)					6.96 ± 0.85	6.74 ± 1.03	7.41 ± 0.97	7.32 ± 1.05	7.90 ± 0.93^‡‡^	7.52 ± 0.91	7.94 ± 0.86^‡‡^	7.60 ± 1.04	0.342
Systemic oxygen consumption (mL O_2_/kg/min)					2.04 ± 1.24	2.20 ± 1.00	2.78 ± 0.89	1.93 ± 0.57	2.29 ± 1.02	2.05 ± 0.47	2.20 ± 0.51	2.13 ± 0.38	0.282

Data are expressed as mean ± SD. CPB 1, CPB 2, CPB 3, and CPB 4 represent consecutive 30-min experimental periods during CPB. *n* = 7 for both vehicle (saline) and dexmedetomidine (Dex) groups. Between-group and within-group comparisons during the interventional periods (anaesthesia to the end of CPB) were made using a two-way or mixed-effects model (if there is missing data) repeated measures analysis of variance (ANOVA).

^‡^*p* ≤ 0.05, ^‡‡^*p* ≤ 0.01, ^‡‡‡^*p* ≤ 0.001, within-group comparison between experimental periods using Tukey’s post-test. *p*_Interaction_ represents treatment–time interaction between groups from a two-way ANOVA analysis

### *In vivo *renal macro- and micro-circulatory changes

Dexmedetomidine did not affect renal blood flow, renal vascular conductance, renal delivery of oxygen and renal oxygen consumption significantly during CPB (Supplementary Fig. 1). Renal cortical and medullary perfusion and oxygenation were not significantly different between the vehicle and dexmedetomidine groups at any time point (*p*_Group_ > 0.05, Supplementary Fig. 2A–D). Renal functional outcomes, urine output, plasma creatinine and sodium excretion, creatinine clearance, total sodium reabsorption and fractional excretion were all similar between the vehicle and dexmedetomidine groups except during anaesthesia where the dexmedetomidine-treated group showed significantly increased creatinine clearance (*p* = 0.019) and sodium reabsorption (*p* = 0.022, Supplementary Table 1).

### Renal interlobar artery contractility

**Fig. 2:**
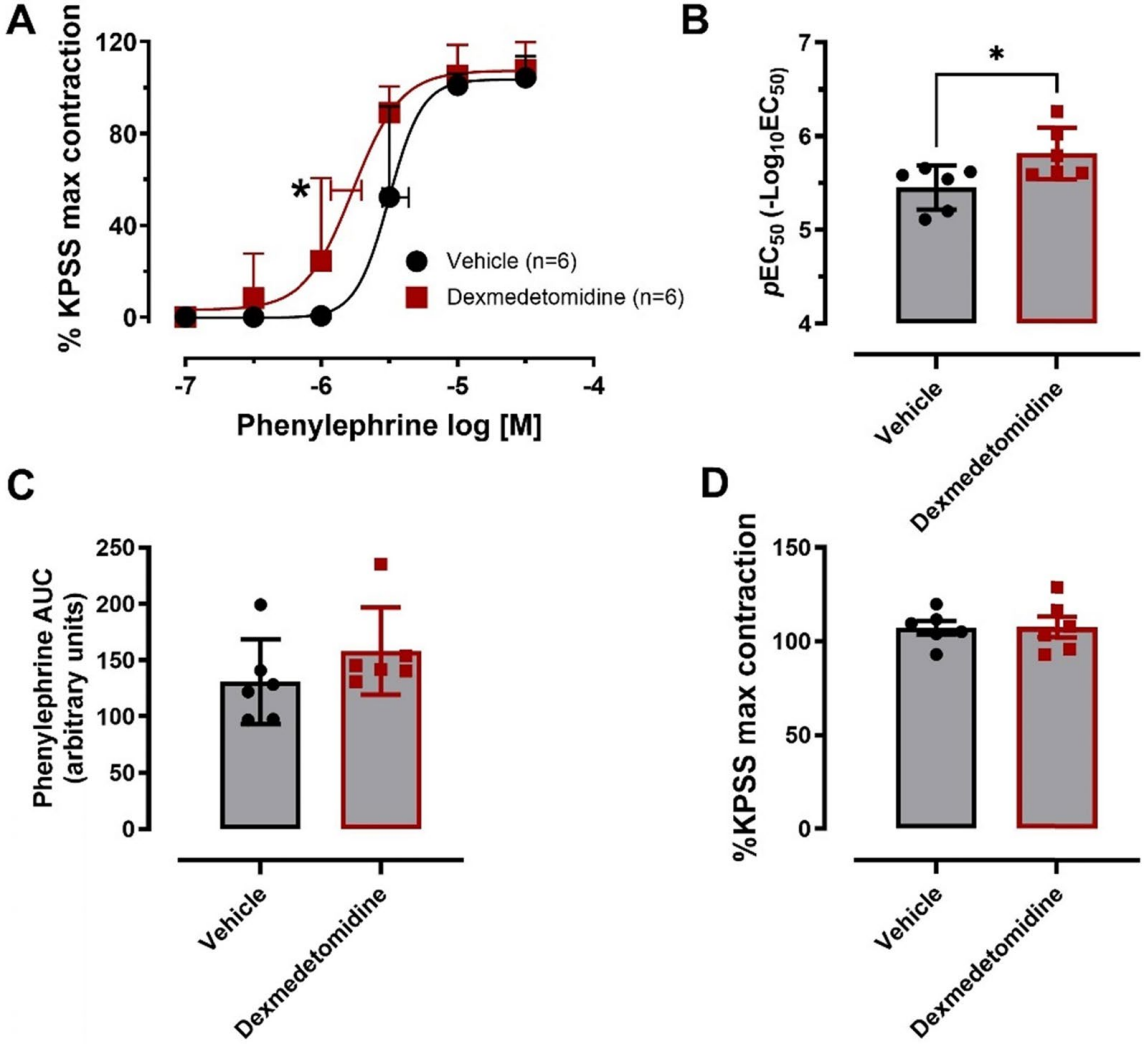
Vascular contractile response of sheep isolated renal interlobar arteries to phenylephrine *in vitro*. **A** Concentration-dependent contractile responses of renal interlobar arteries to phenylephrine. **B** The potency of the phenylephrine contractile responses expressed as *p*EC50 (−log EC50). **C** The area under the phenylephrine contractile responses and **D** the maximal contraction at the highest concentration tested (100 µM). Error bars are SD (those not shown are contained within the symbol); horizontal error bars are for the average EC_50_ ± SEM. n, number of arteries isolated from separate sheep. *p ≤ 0.05, two-tailed unpaired Student’s *t*-test

Phenylephrine caused concentration-dependent contractions in both groups (Fig. 2A). The potency of the α_1_-agonist, phenylephrine, was 2.3-fold greater in the arteries from sheep that received dexmedetomidine, relative to those treated with vehicle (*p*EC_50_: 5.82 ± 0.27 vs. 5.45 ± 0.23 in vehicle groups, *p* = 0.034, unpaired t-test, Fig. 2A, 2B). The area under the phenylephrine concentration response curve showed a trend to increase in the dexmedetomidine group (Fig. 2C). Conversely, at high concentrations of phenylephrine (10–30 µM), there were no significant differences between groups. The maximal contractile responses to phenylephrine were similar in the vehicle (107 ± 9%) and dexmedetomidine groups (108 ± 13%, Fig. 2D). Data from 2 sheep were excluded due to the requirement of high doses of metaraminol (an α_1_-agonist) to maintain blood pressure during CPB, which could make the arteries unresponsive to phenylephrine (tachyphylaxis).

### Renal interlobar artery relaxation

**Fig. 3:**
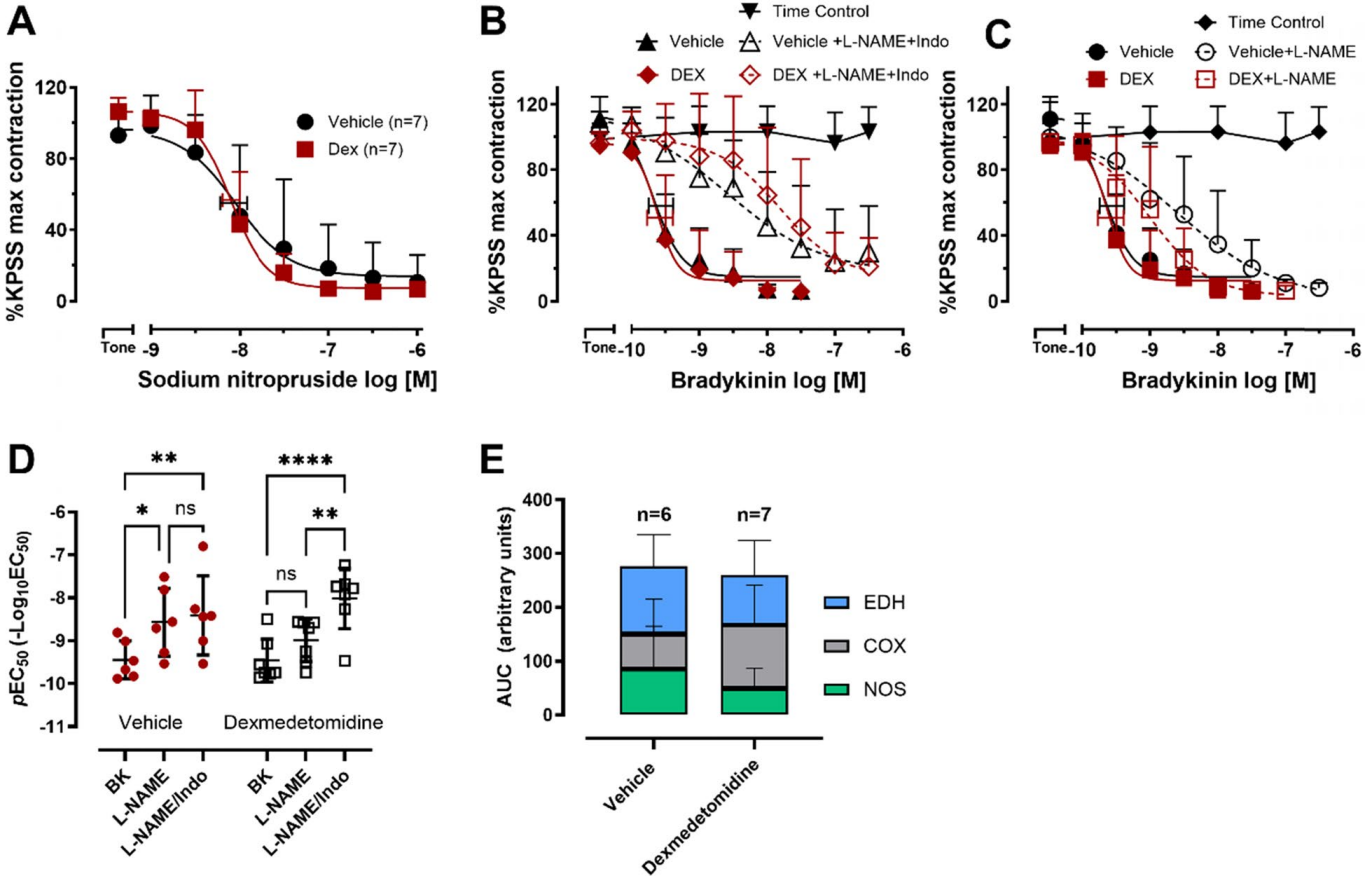
Vascular relaxant response of sheep isolated renal interlobar arteries in vitro. **A** Relaxation responses to the endothelium-independent dilatory sodium nitroprusside. **B** Relaxation of interlobar arteries to the endothelium-dependent dilator bradykinin in the absence (solid circle and square symbols) or presence of the nitric oxide synthase inhibitor L-NAME (200 µM) and cyclooxygenase inhibitor indomethacin (3 µM) (open triangles). **C** Relaxation response to bradykinin in the presence of L-NAME (200 µM). The bradykinin response in the absence of any inhibitor shown in **B** is also shown in **C** for convenience. Time control refers to arteries left at the contractile tone without the addition of any relaxant agent to assess time-dependent decrease in tone. **E** The log EC_50_ of each group. **F** The area under the bradykinin curve showing the relative contribution of nitric oxide synthase (NOS), cyclooxygenase (COX) and endothelium-dependent hyperpolarisation (EDH) pathways to the bradykinin relaxation. **A**–**D** Data are presented as a % of the KPSS (100 mM K^+^)-evoked contractions. Error bars are SD (those not shown are contained within the symbol); horizontal error bars are for the average EC_50_ ± SEM. n, number of arteries isolated from separate sheep. DEX; dexmedetomidine. ^#^*p* ≤ 0.05, two-tailed unpaired Student’s *t*-test; *p ≤ 0.05, **p ≤ 0.01, ***p ≤ 0.001, two-way ANOVA with Sidak’s post-test. ns, not statistically significant

The endothelium-independent relaxant sodium nitroprusside elicited a concentration-dependent relaxation of isolated renal interlobar arteries in both groups (Fig. 3A). The potency (*p*EC_50_: 7.97 ± 0.39 in vehicle and 8.09 ± 0.26 in Dex groups) and efficacy (88 ± 18% and 95 ± 3% relaxation from baseline contraction in vehicle and Dex groups, respectively) of sodium nitroprusside was similar between the groups (*p* = 0.492, unpaired *t*-test).

The maximum endothelium-dependent relaxation elicited by bradykinin from the baseline contraction was similar in the vehicle (93 ± 3% relaxation from baseline contraction) and dexmedetomidine groups (93 ± 2% relaxation from baseline contraction, Fig. 3B). The potency of bradykinin relaxation was also comparable (*p*EC_50_: 9.45 ± 0.44 in vehicle and 9.46 ± 0.50 in dexmedetomidine groups).

To investigate differences in the contribution of the different vasodilatory pathways to the endothelium-dependent relaxation by bradykinin, separate groups of arteries were pretreated with either a nitric oxide synthase (NOS) inhibitor (L-NAME, 200 µM) or in combination with a cyclooxygenase inhibitor (indomethacin, 3 µM) and a NOS inhibitor (L-NAME, 200 µM). The relaxation remaining after treatment with indomethacin and L-NAME was attributed to endothelium-dependent hyperpolarisation (EDH). The NOS inhibitor, L-NAME, caused a significant decrease in potency of the bradykinin relaxation in the vehicle group (sevenfold shift to *p*EC_50_ 8.57 ± 0.79, *p* = 0.014, two-way ANOVA with Sidak’s post-test) but only showed a trend in the dexmedetomidine group (threefold shift to *p*EC_50_ 8.99 ± 0.49,* p* = 0.192 Fig. 3C, 3E). The addition of the cyclooxygenase inhibitor, indomethacin, together with NOS inhibition caused a significant decrease in the bradykinin relaxation potency compared with the absence of the inhibitors in both the vehicle (11-fold shift to *p*EC_50_ 8.38 ± 0.36,* p* = 0.004) and dexmedetomidine groups (28-fold shift to *p*EC_50_ 8.02 ± 0.27, *p* < 0.0001, two-way ANOVA, Fig. 3D, 3E). To assess the specific contribution of the cyclooxygenase pathway, we compared the potency of bradykinin in the presence of L-NAME alone with the combination of L-NAME and indomethacin. Only the dexmedetomidine-treated group showed a significant further rightward shift in the relaxation to bradykinin with the addition of indomethacin (ninefold shift from *p*EC_50_ 8.99 ± 0.19 to 8.02 ± 0.27, *p* = 0.004, two-way ANOVA with Sidak’s post-test, Fig. 3D**, **Fig. 3E). Although there were differences in the contribution of the vasodilatory pathways to each individual group, there was no difference observed between the vehicle and dexmedetomidine groups when comparing each pathway across the groups (*p*_Group_ and *p*_Interaction_ > 0.05, two-way ANOVA).

The contribution of each dilatory pathway to the efficacy of bradykinin was further assessed by using the area under the bradykinin relaxation curve (AUC) in the absence and presence of inhibitors. In the vehicle group, the endothelium-dependent hyperpolarisation pathway contributed the most (45 ± 21%) followed by the NOS pathway (31 ± 28%) and COX pathway (23 ± 23%), as opposed to the dexmedetomidine group where the COX pathway contributed the most (45 ± 28%) and the NOS pathway contributed the least (19 ± 14%) (Fig. 3F). There was no statistically significant difference between the vehicle and dexmedetomidine groups (*p*_Group_ and *p*_Interaction_ > 0.05, two-way ANOVA).

### Histopathological analysis of renal interlobar artery endothelial cells

**Fig. 4:**
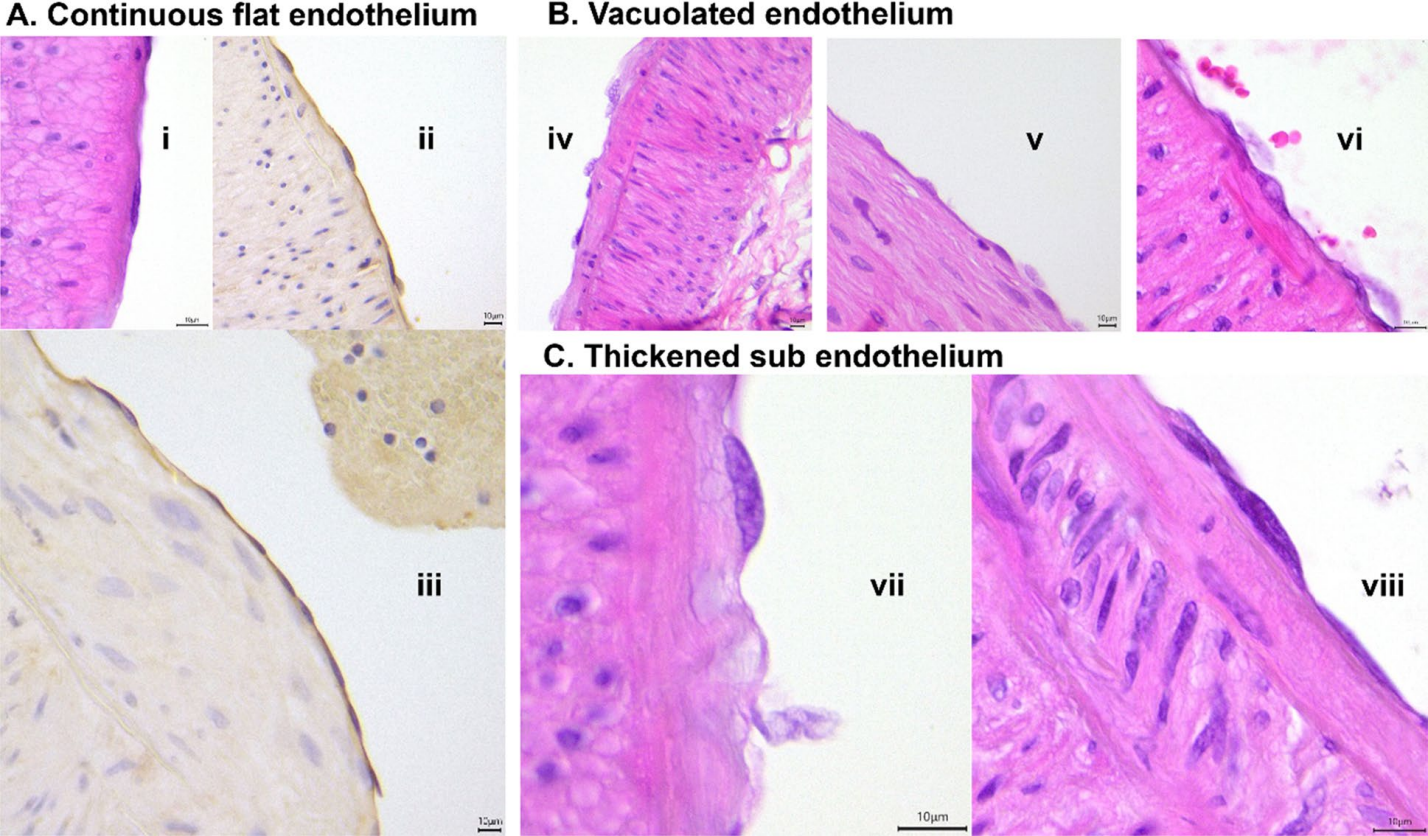
Representative images of the endothelial lining of sheep isolated renal interlobar arteries. **A** Shows continuous flat endothelium (i, ii, iii), **B** shows vacuolated endothelium (iv, v, vi) and **C** shows a thickened sub-endothelium (vii, viii). ii and iii are from staining with *Datura stramonium* L and the rest are from H&E staining. Scale bars are 10 µm

Endothelial cell morphology in both the vehicle and dexmedetomidine groups was normal with most sections assessed showing continuous, flat endothelial cells similar to healthy controls (Table 2). Vacuolation within the endothelial cells was observed in 2/7 sheep in the vehicle and 4/7 sheep in the dexmedetomidine groups, but a similar finding was seen in renal interlobar artery endothelial cells obtained from healthy controls (4/7 sheep) (Table 2**, **Fig. 4), suggesting this may be a common occurrence in sheep interlobar arteries or an artefact of the technique employed. There was no statistical difference in the degree of vacuolation between vehicle and dexmedetomidine groups using the Mann–Whitney test or Fisher’s exact test (Table 2). Thickened sub-endothelium was observed in the endothelial layer of one sheep (CPB85) from the vehicle group and one (A14) from the healthy controls. No other obvious histopathological changes were observed. Assessment of oxidative stress in renal artery homogenate using the lipid peroxidation marker malondialdehyde (MDA) showed that there were no differences between the dexmedetomidine (4.55 ± 0.72 nmol/mg protein) and vehicle (4.62 ± 0.76 nmol/mg protein) groups (Supplementary Fig. 3).

**Table 2:** Renal interlobar artery endothelial cell semiquantitative histopathological assessment in CPB (vehicle and dexmedetomidine treated) and healthy groups

Histopathology	CPB vehicle (saline)							CPB dexmedetomidine							Healthy controls						
Animal ID	65	68	70	77	81	83	85	69	71	72	75	78	79	86	A11	A14	H1	H2	H3	H4	H5
Flat continuous endothelium	0	0	0	0	0	0	0	0	0	0	0	0	0	0	0	0	0	0	0	0	0
Vacuolation in endothelium	0	0	0	0	+	+	0	+ +	0	+ +	0	+	+ +	0	0	+ +	+	+	0	+	0

Zero (0) no histological changes; ( +) mild change and (+ +) moderate change

## Discussion

In this randomised, placebo-controlled trial, we investigated potential vascular mechanisms by which dexmedetomidine may cause renal tubular injury [11]. Using an established ovine model of CPB, we found that dexmedetomidine, compared with placebo, resulted in increased renal lobar arterial responsiveness to the α_1-_ adrenergic agonist, phenylephrine. This occurred despite no between-group changes in renal oxygen delivery or renal blood flow. However, it is biologically plausible that the observed changes may contribute to focal ischaemia by inducing microcirculatory dysfunction that is not detectable by measurements of global or regional kidney perfusion or oxygenation. Our results are consistent with other studies that reported the peripheral vascular haemodynamic effects of intravenous dexmedetomidine [12–15, 28, 29]. Perioperative dexmedetomidine had no observable effects on renal vascular endothelial-dependent or -independent relaxation nor on endothelial histopathology following ovine CPB.

Vasopressors are administered frequently during and after CPB to treat vasoplegia, maintain target MAP and thereby ensure adequate perfusion [21, 22, 30]. Agents such as phenylephrine and noradrenaline, which act primarily through α₁-adrenergic receptor stimulation, are often favoured due to their potent peripheral vasoconstrictive properties [21, 22]. However, while MAP may be maintained, intense vasoconstriction in the renal vasculature, particularly in preglomerular resistance vessels, can lead to a mismatch between oxygen delivery and metabolic demand [31, 32]. The renal medulla, which has a reduced autoregulatory capacity compared with the renal cortex under normal physiological conditions [31, 33], is especially susceptible to reduced perfusion and oxygenation. Catecholamine vasopressors have been documented to compromise renal medullary perfusion and oxygenation selectively in healthy sheep [31] and those with sepsis [34]. We have recently reported that norepinephrine further exacerbates the incipient renal medullary tissue hypoxia in ovine CPB, an effect not observed during the maintenance of target MAP with metaraminol, phenylephrine or vasopressin [35]. When combined with agents like dexmedetomidine that have been shown to augment α₁-mediated vasoreactivity, the risk of regional hypoperfusion, hypoxia and consequent tubular injury may be amplified. Thus, while vasopressors are essential for haemodynamic stability in low afterload states, their renal effects warrant careful consideration, particularly in the setting of CPB where kidneys are highly vulnerable to ischaemic and hypoxic insults [36].

We found that isolated renal resistance arteries demonstrate augmented contractile responsiveness to phenylephrine in response to dexmedetomidine prior to and during CPB in sheep. Enhanced responsiveness to α₁-adrenergic agonists, such as phenylephrine, can lead to excessive vasoconstriction in preglomerular vessels, including the renal lobar and interlobar arteries. Similarly, Yildiz et al. [15] reported that dexmedetomidine caused a direct α_2_-mediated vasoconstriction, with activation of α_1_-adrenoceptors at higher concentrations in human isolated internal mammary arteries. In the context of cardiac surgery and CPB, where renal perfusion is already vulnerable to systemic haemodynamic fluctuations, an exaggerated vasoconstrictive response may focally reduce renal tissue perfusion [37] that could impair oxygen delivery to tubular segments with high metabolic demand, resulting in mitochondrial dysfunction, ATP depletion, and ultimately, tubular epithelial injury or death [38]. This mechanism may be particularly relevant when dexmedetomidine is co-administered with α₁-agonists to support systemic blood pressure perioperatively, inadvertently exacerbating renal microvascular dysregulation. These findings underscore the importance of carefully balancing vasopressor therapy and dexmedetomidine use to minimise the risk of renal ischaemic tubular injury during cardiac surgery.

Histological analysis further confirmed that dexmedetomidine did not induce pathological changes in the endothelium of renal interlobar arteries. Despite the enhanced vasoconstrictor responsiveness of isolated interlobar arteries, dexmedetomidine had no measurable impact on whole-kidney oxygen delivery, consumption, or microcirculatory perfusion and oxygenation relative to placebo, confirming the previous report by Jufar et al*.* [11]. A plausible explanation for this apparent disconnect is a redistribution of sodium reabsorption from metabolically efficient nephron segments, such as the thick ascending limb of the loop of Henle, to less efficient segments, such as the collecting ducts [39, 40]. Renal tissue oxygenation is tightly regulated by the balance between local oxygen delivery and consumption. While oxygen delivery can be inferred from blood oxygen content and perfusion measurements, it is technically challenging to quantify regional oxygen consumption directly. No validated methods exist currently to measure oxygen consumption at the microregional level in the kidney; this is acknowledged as a significant limitation in renal oxygenation research. In the context of our study, this prevents us from drawing definitive mechanistic conclusions regarding dexmedetomidine-associated tubular injury.

Dexmedetomidine did not affect the relaxation responses of renal interlobar arteries to either endothelium-dependent or endothelium-independent vasodilators *in vitro*, compared with placebo. Notably, the vasodilatory response to bradykinin shifted from being chiefly mediated by nitric oxide synthase to a cyclooxygenase-dependent pathway. These findings are consistent with those of previous studies in which no direct endothelial effects of dexmedetomidine in arteries with intact endothelium were found [41].

Dexmedetomidine is reported to have organ protective effects in adult and paediatric cardiac and non-cardiac surgery populations (see meta-analyses [9, 42]) at moderate doses due to the wide expression of α_2_-adrenoceptors in the liver, kidneys, lungs and brain. Some of the suggested mechanisms involve an anti-inflammatory effect, improved renal blood flow, preserved glomerular filtration, increased urinary output, and decreased blood urea nitrogen, creatinine and renin levels [42–45]. Some studies report no therapeutic effect or risk signals particularly due to bradycardia, hypotension and mismatch of blood flow at the microcirculatory level [10, 11, 46]. We speculate that the histopathological tubular injury that we [11] and others [10] have reported may be associated with increased contractility of the microvasculature locally. Many clinical trials use a range of doses, some with a loading dose (0.4–1.0 μg/kg) and others with only a continuous infusion (0.04–1.5 μg/kg/h) from the induction of general anaesthesia to the end of surgery or up to 24 h postoperatively, making quantitative assessment of clinical pharmacokinetics and vasoconstriction difficult [9, 10, 29]. Our findings suggest that future trials should be designed to assess the best choice of vasoconstrictors used during CPB where dexmedetomidine is employed.

Our study has several strengths. We used a clinically relevant large animal model that used strategies that closely mimic the current recommendations for human CPB, including the conduct of anaesthesia, target perfusion pressure, pump flow, temperature and anticoagulation [11, 22, 47, 48]. Our ovine model allows us to instrument the kidney and brain extensively and collect tissue immediately postmortem to enable a more detailed mechanistic investigation than is possible in humans. The dose of dexmedetomidine infusion was the same as that used in many clinical trials. To our knowledge, this is the first study to assess isolated renal vascular function after CPB.

We acknowledge some limitations. First, we used young healthy sheep in contrast to patients undergoing cardiac surgery who are older with multiple medical comorbidities such as hypertension, diabetes, heart failure and/or chronic kidney disease. Therefore, future studies that incorporate comorbid animal models are needed. Second, in this study, we used only female sheep. This decision reflects practical and ethical constraints associated with our large animal model: male rams are substantially more aggressive and pose significant occupational safety risks, cannot be housed in the same facility as female sheep, and prohibits urethral bladder catheterisation. Urethral catheterisation required for continuous urine output monitoring is technically unfeasible in male sheep due to their long, sigmoid urethra. While the use of female animals ensures procedural feasibility and operator safety, we acknowledge that this sex restriction may limit the generalisability of our findings. Third, our study did not examine the effects of dexmedetomidine after CPB, while many trials continued the infusion up to 24 h postoperatively. This is important as most clinical studies that found reduced incidence of AKI used a longer duration of treatment. Fourth, we employed direct measures of perfusion and oxygenation using single probes that only provide localised measurements rather than global renal cortical or medullary ischaemia. Fifth, only researchers performing the histopathological assessment but not the in vivo physiological measurements were blinded to the group allocation. Finally, we acknowledge that several perioperative factors inherent to CPB including the anaesthetic, analgesic and vasoactive regimens, mild hypothermia, and the systemic inflammatory response may themselves contribute to renal microvascular alterations. While these variables were standardised across animals in dexmedetomidine compared with vehicle treatment and reflect contemporary clinical practice, our study was not designed to isolate their individual effects. Consequently, we cannot fully disentangle the contribution of these perioperative factors from the vascular and endothelial changes observed. Future studies incorporating factorial or mechanistic designs are warranted to dissect the relative impact of these potential confounders.

## Conclusion

In this randomised controlled trial using a clinically relevant ovine model of CPB, we identified a potential vascular mechanism by which dexmedetomidine may contribute to renal tubular injury. Specifically, dexmedetomidine enhanced renal lobar arterial responsiveness to the α₁-adrenergic agonist phenylephrine, suggesting that its use may exacerbate vasoconstriction and promote focal renal ischaemia when administered perioperatively to maintain perfusion pressure. Importantly, dexmedetomidine did not impair endothelium-dependent or independent vasorelaxation, nor did it induce histopathological injury to the renal vascular endothelium and did not cause a change in the oxidative stress marker MDA during CPB. Collectively, our results suggest that while dexmedetomidine does not cause global endothelial dysfunction, its potentiation of α₁-mediated vasoconstriction may contribute to local disturbances in renal perfusion, thereby increasing the risk of tubular injury. Further studies are warranted to elucidate additional mechanisms of dexmedetomidine-induced renal injury and to inform safer perioperative use of this agent in cardiac surgery.

## Supplementary Information

Additional file 1.

## Data Availability

All data generated or analysed during this study are included in this published article and its supplementary information files. Datasets are available from the corresponding author upon request.
