# Supplementary material for: Effects of perioperative dexmedetomidine on renal vascular function and renovascular histopathology in ovine cardiopulmonary bypass
Source: Intensive Care Med Exp. 2025 Dec 16;13:128. doi: 10.1186/s40635-025-00836-7 (PMC12705920; doi:10.1186/s40635-025-00836-7)
Supplement: Supplementary file 1 — Additional file 1. [file 40635_2025_836_MOESM1_ESM.docx]

**Supplementary Information**

**Effects of perioperative dexmedetomidine on renal vascular function and renovascular histopathology in ovine cardiopulmonary bypass**

Ashenafi H. Betrie^1,2^, Alemayehu H. Jufar^1^, Roger G. Evans^1,3^, Andrew D. Cochrane^1,4^, Bruno Marino^5†^, Ian Birchall^1,6^, Sally G. Hood^1^, Peter R. McCall^7^, Scott Ayton^2^, Lachlan F. Miles^1,7,8^, Clive N. May^1,8^, Yugeesh R. Lankadeva^1,7,8,*^

*^1^Florey Institute of Neuroscience and Mental Health, The University of Melbourne, Victoria, Australia*

*^2^Florey Department of Neuroscience and Mental Health, The University of Melbourne, Victoria, Australia*

*^3^Biomedicine Discovery Institute and Department of Physiology, Monash University, Melbourne, Victoria, Australia,*

*^4^Department of Cardiothoracic Surgery, Monash Health and Department of Surgery, Monash University, Melbourne, Victoria, Australia*

*^5^Cellsaving and Perfusion Resources, Melbourne, Victoria, Australia.*

*^6^Department of Surgery, The University of Melbourne, Victoria, Australia*

*^7^Department of Anaesthesia, Austin Hospital, Melbourne, Australia*

*^8^Department of Critical Care, Melbourne Medical School, The University of Melbourne, Victoria, Australia*

^†^ Author deceased prior to publication

***Correspondence to:**

Professor Yugeesh R Lankadeva

Translational Cardiovascular and Renal Research Group, The Florey Institute of Neuroscience and Mental Health

30 Royal Parade, Parkville Victoria 3052, Australia.

Tel: +61406649976

E-mail: [yugeesh.lankadeva@florey.edu.au](mailto:yugeesh.lankadeva@florey.edu.au)

**Supplementary Results**

**In vivo renal macro- and microcirculatory changes**

Renal blood flow, renal vascular conductance, renal delivery of oxygen and renal oxygen consumption decreased significantly during anaesthesia and CPB compared with levels in conscious sheep in both the vehicle and dexmedetomidine groups (*p*_Time_<0.0001). There were, however, no differences between the groups at each time point; conscious, anaesthesia or CPB except for renal delivery of oxygen where the dexmedetomidine group was significantly less than the vehicle group during anaesthesia (*p*=0.023) (**Supplementary Fig 1**).

Renal medullary perfusion and oxygenation were not different between the vehicle and dexmedetomidine groups at any of the time points (*p*_Group_>0.05, **Supplementary Fig 2A, 2B**). In the vehicle group, renal medullary perfusion decreased significantly during anaesthesia (702 ± 140 BPU, *p*=0.003) and CPB (441 ± 74 BPU, *p*=0.011) compared with the levels in the conscious state (801 ± 154 BPU), but not in the dexmedetomidine group (*p*_Time_=0.0002, **Supplementary Fig 2A**). Similarly, there was significant increase in medullary oxygenation during anaesthesia in the vehicle group (from 39 ± 8 to 47 ± 12 mmHg; *p*<0.0001) and in the dexmedetomidine group (from 36 ± 8 mmHg to 43 ± 15 mmHg, *p*=0.008) compared with the levels in the conscious period. During CPB, medullary oxygenation decreased in both the vehicle (to 11 ± 6 mmHg, *p*=0.008) and dexmedetomidine groups (to 11 ± 10 mmHg, *p*=0.011, **Supplementary Fig 2B**) compared with the conscious values.

Renal cortical perfusion only showed a trend to decrease during anaesthesia in both the vehicle (from 1327 ± 212 to 971 ± 154 BPU, *p*=0.052) and dexmedetomidine groups (from 1375 ± 114 to 1029 ± 376 BPU, *p*=0.086) compared with the levels in the conscious state (*p*_Time_<0.0001). But during the last 30 min of CPB, there was a significant decrease in both the vehicle (760 ± 157 BPU, *p*=0.01) and dexmedetomidine groups (588 ± 143 BPU, *p*=0.002, **Supplementary Fig 2C**) compared with the levels in the conscious state. Renal cortical oxygenation showed a significant increase during anaesthesia in the vehicle (from 46 ± 7 mmHg to 66 ± 7 mmHg, *p*=0.003) and dexmedetomidine groups (from 40 ± 12 mmHg to 67 ± 23 mmHg, *p*=0.011), but during CPB it returned to the levels in the conscious state (*p*_Time_=0.002, **Supplementary Fig 2D**). There were no statistically significant differences in renal cortical perfusion or oxygenation between the groups at any of the time points; conscious, anaesthesia or CPB.


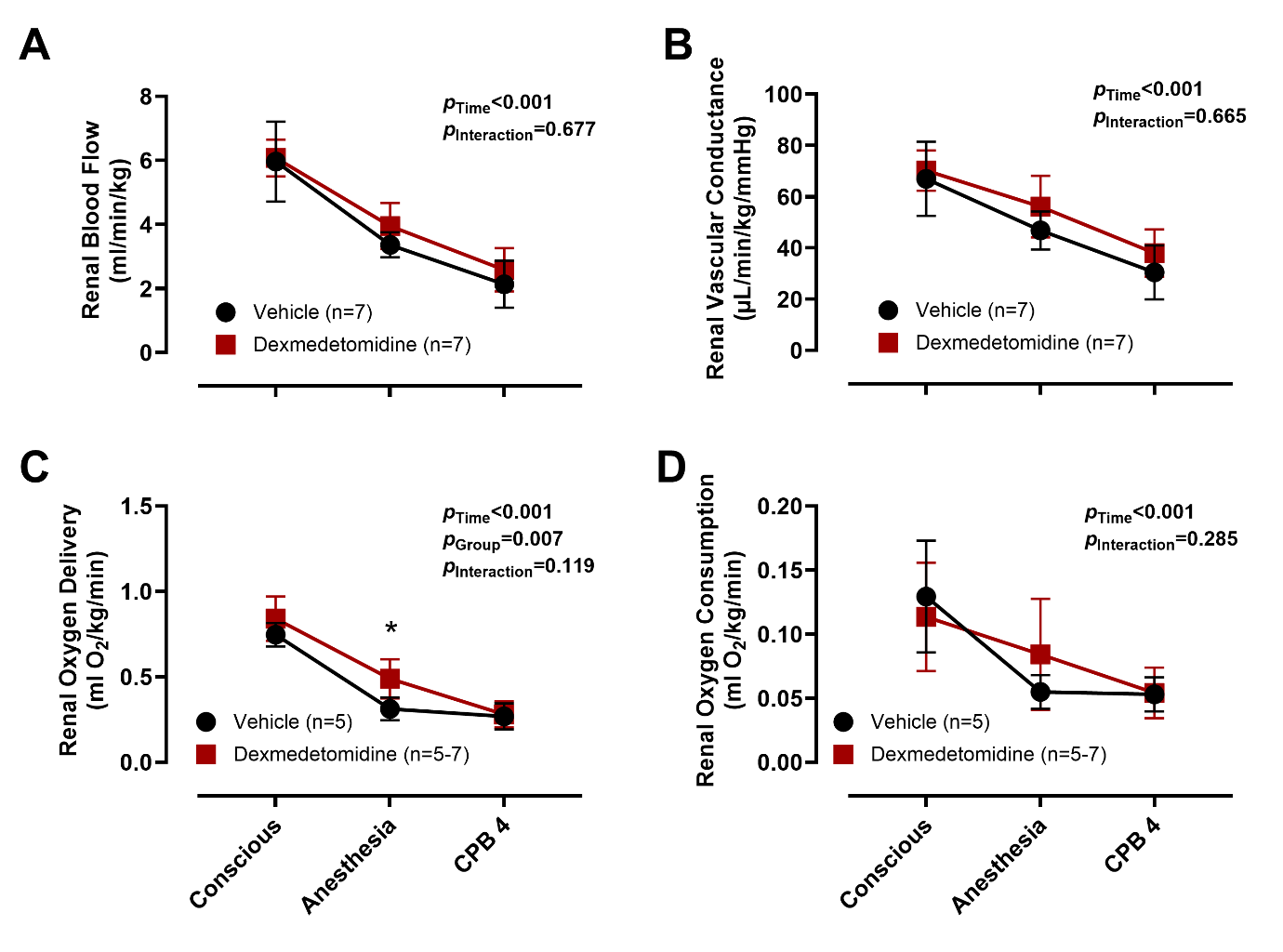


**Supplementary Fig 1: Renal macrocirculatory changes in an ovine model of CPB (*in vivo)*.** (A) Renal blood flow, (B) renal vascular conductance, (C) oxygen delivery and (D) oxygen consumption during the conscious baseline state, under anaesthesia and during the last 30 min of CPB for 2-h (CPB4) in an ovine model. Between-group and within-group comparisons were made using a two-way or mixed-effects model (if there was missing data) repeated measures analysis of variance (ANOVA). Data are expressed as mean ± SD, n is number of animals. ^*^*p*≤0.05, Tukey’s post-test group comparison between vehicle (saline) and dexmedetomidine. *p*_Interaction_ represents treatment-time interaction between groups from a two-way ANOVA analysis.


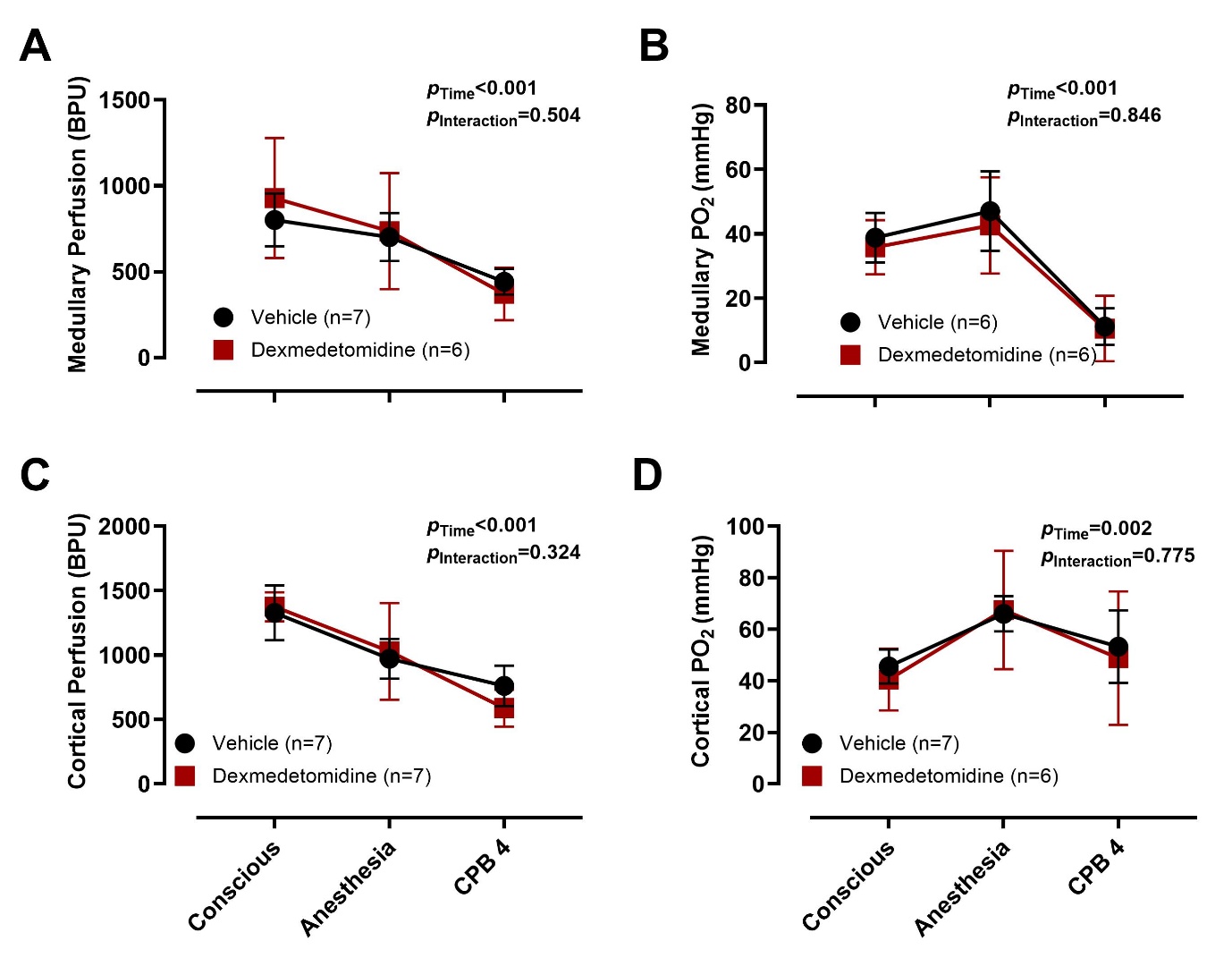


**Supplementary Fig 2: Renal microcirculatory changes in an ovine model of CPB (*in vivo)*.** (A) Renal medullary perfusion, (B) oxygenation and renal cortical perfusion (C) and oxygenation (D) at conscious baseline, during anaesthesia and the last 30 minutes of a 2-h CPB (CPB4) in ovine model receiving vehicle (saline) or dexmedetomidine. Data expressed as mean ± SD, n is number of animals. Between-group and within-group comparisons were made using a two-way or mixed-effects model (if there is missing data) repeated measures analysis of variance (ANOVA).


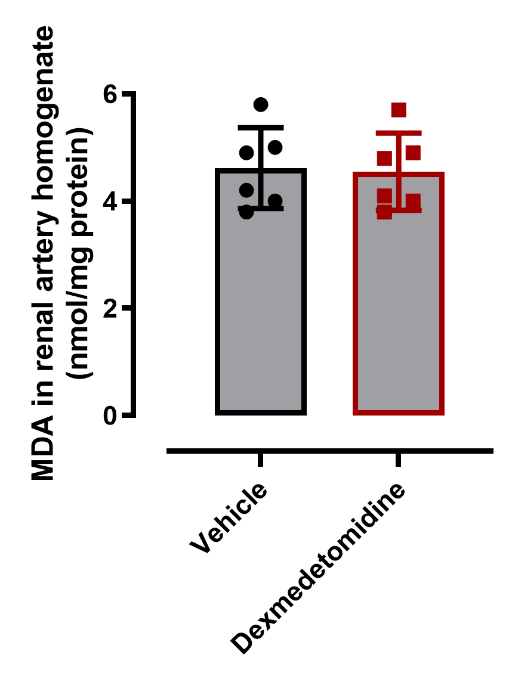


**Supplementary Fig 3: Malondialdehyde (MDA) levels in renal interlobar artery homogenate.**

MDA levels were assessed by lipid peroxidation assay (Abcam, ab118970) using 100 ug of protein from homogenized renal artery lysate and fluorescence (excitation 532 nm, emission 553 nm) was changes were measured with a CLARIOstar plate reader.

**Supplementary Table** **1. Summary of renal functional outcomes in sheep treated with dexmedetomidine or vehicle during cardiopulmonary bypass.**

| **Variable** | **Conscious** | | **Anaesthetised** | | **Cardiopulmonary bypass (CPB)** | | | | | | | | | |
| --- | --- | --- | --- | --- | --- | --- | --- | --- | --- | --- | --- | --- | --- | --- |
|  | **Vehicle** | **Dex** | **Vehicle** | **Dex** | **Vehicle CPB 1** | **Dex**  **CPB 1** | **Vehicle CPB 2** | **Dex**  **CPB 2** | **Vehicle CPB 3** | **Dex**  **CPB 3** | **Vehicle CPB 4** | **Dex**  **CPB 4** | ***P*_Interaction_** |  |
| Plasma Creatinine (µmol/L) | 69.7 ± 10.3 | 70.6 ± 5.6 | 73.1 ± 13.9 | 65.0 ± 4.5 | 69.1 ± 12.3 | 62.6 ± 2.5 | 72.4 ± 12.7 | 64.2 ± 1.8 | 72.4 ± 13.6 | 64.6 ± 2.9 | 72.7 ± 14.8 | 64.8 ± 3.1 | 0.273 |  |
| Urine Flow  (mL/kg/h) | 1.9 ± 1.3 | 1.5 ± 1.4 | 0.5 ± 0.2 | 1.3 ± 0.9 | 4.4 ± 1.6 | 5.6 ± 1.6 | 2.8 ± 1.3 | 3.6 ± 2.7 | 2.1 ± 0.7 | 3.0 ± 2.7 | 1.5 ± 0.7 | 3.0 ± 3.0 | 0.472 |  |
| Sodium Excretion  (µmol/kg/min) | 2.97 ± 1.72 | 2.07 ± 2.72 | 1.12 ±1.45 | 2.97 ± 3.27 | 8.06 ± 5.03 | 9.76 ± 3.65 | 4.82 ± 3.70 | 7.08 ± 4.47 | 2.91 ± 1.94 | 4.85 ± 3.35 | 1.93 ± 1.30 | 2.85 ± 2.21 | 0.515 |  |
| CrCl  (mL/kg/min) | 1.93 ± 0.93 | 1.67 ±1.26 | 0.93 ± 0.51 | 1.59 ±0.31**^‡^** | 1.52 ± 0.53 | 1.62 ± 0.61 | 1.01 ± 0.42 | 1.38 ± 0.50 | 0.91 ± 0.39 | 1.25 ± 0.38 | 0.75 ± 0.35 | 1.11 ± 0.47 | 0.335 |  |
| T_Na+_  (µmol/kg/min) | 276.4 ± 135.1 | 240.1 ± 181.7 | 133.7 ± 73.6 | 226.2 ± 44.1**^‡^** | 210.1 ± 77.6 | 222.8 ± 84.9 | 140.2 ± 59.3 | 190.8 ± 66.8 | 127.7 ± 56.5 | 174.0 ± 50.1 | 105.3 ± 48.8 | 157.3 ± 64.9 | 0.352 |  |
| Fractional Excretion of Sodium (%) | 1.16 ± 0.74 | 0.80 ± 0.61 | 0.88 ±0.50 | 1.25 ± 1.25 | 4.24 ± 2.97 | 4.71 ± 2.75 | 3.31 ± 2.17 | 3.39 ± 1.39 | 2.68 ± 2.46 | 2.51 ± 1.22 | 2.08 ± 1.55 | 1.79 ±1.07 | 0.829 |  |

Data are expressed as mean ± SD. CPB 1, CPB 2, CPB 3, and CPB 4 represent consecutive 30-minute experimental periods during CPB. n=7 for both vehicle and dexmedetomidine (Dex) groups for Urine flow but for the rest of the variables, n=7 for vehicle and n=5 for Dex groups. Creatinine clearance (CrCl) is the product of urine flow and concentration of creatine in the urine divided by concentration of creatinine in the plasma. Sodium reabsorption (T_Na+_) is the product of CrCl and the concentration of sodium in the plasma, minus sodium excretion. Between-group and within-group comparisons during the interventional periods (anaesthesia to the end of CPB) were made using a two-way or mixed-effects model (if there is missing data) repeated measures analysis of variance (ANOVA). ^‡^*p* ≤0.05, Across group comparison between experimental groups using Tukey’s post-test. *P*_Interaction_ represents treatment-time interaction between groups from a two-way ANOVA analysis.
